# Performance Evaluation of IMU and DVL Integration in Marine Navigation

**DOI:** 10.3390/s21041056

**Published:** 2021-02-04

**Authors:** Gen Fukuda, Daisuke Hatta, Xiaoliang Guo, Nobuaki Kubo

**Affiliations:** 1 Department of Maritime Systems Engineering, Tokyo University of Marine Science and Technology, Tokyo 135-8533, Japan; nkubo@kaiyodai.ac.jp; 2 Maritime Technology and Logistics, Tokyo University of Marine Science and Technology, Tokyo 135-8533, Japan; m195015@edu.kaiyodai.ac.jp (D.H.); m195022@edu.kaiyodai.ac.jp (X.G.)

**Keywords:** inertial measurement unit, Doppler velocity log, global navigation satellite system, marine navigation

## Abstract

Global navigation satellite system (GNSS) spoofing poses a significant threat to maritime logistics. Many maritime electronic devices rely on GNSS time, positioning, and speed for safe vessel operation. In this study, inertial measurement unit (IMU) and Doppler velocity log (DVL) devices, which are important in the event of GNSS spoofing or outage, are considered in conventional navigation. A velocity integration method using IMU and DVL in terms of dead-reckoning is investigated in this study. GNSS has been widely used for ship navigation, but IMU, DVL, or combined IMU and DVL navigation have received little attention. Military-grade sensors are very expensive and generally cannot be utilized in smaller vessels. Therefore, this study focuses on the use of consumer-grade sensors. First, the performance of a micro electromechanical system (MEMS)-based yaw rate angle with DVL was evaluated using 60 min of raw data for a 50 m-long ship located in Tokyo Bay. Second, the performance of an IMU-MEMS using three gyroscopes and three accelerometers with DVL was evaluated using the same dataset. A gyrocompass, which is equipped on the ship, is used as a heading reference. The results proved that both methods could achieve less than 1 km horizontal error in 60 min.

## 1. Introduction

Satellite positioning plays an important role in modern navigation systems. Among the existing advanced navigation sensors, only satellite navigation can provide a ship’s absolute position relative to the geocentric coordinate system [1]. When navigating, the marine satellite navigation receiver has an open environment and access to several visible satellites when located in the middle and at low latitudes, resulting in better position accuracy. However, the number of ships lost at sea due to global navigation satellite system (GNSS) spoofing is increasing, according to reports. According to Dryad Global, a maritime security intelligence agency, GNSS interference incidents in the eastern Mediterranean and the Persian Gulf have become more frequent. GNSS interference introduces positioning errors and affects the normal operation of a number of ship’s navigation aids, causing crews to make wrong decisions and posing significant marine security risks [2]. Malicious interference usually occurs near the coast, increasing the risk of vessel grounding.

In the event of GNSS interference, the captain can rely on other navigation sensors. Inertial measurement unit (IMU) and Doppler velocity log (DVL) systems work independently and are not easily disturbed. An IMU provides high-frequency angular velocity and acceleration information; however, it is subject to data bias, which accumulates over time. A high-precision fiber optic gyroscope (FOG) provides reliable long-term navigation calculations [3], but is too expensive for standard use. Micro electromechanical system (MEMS) sensors are cheap, small, and light, and have sufficient accuracy for some applications [4,5]. In offshore areas, a DVL provides accurate information on speed over water and speed over ground, but cannot provide course information [6].

Recently, integrated GNSS and inertial navigation systems (GNSS-INS) have been developed that exploit the accuracy of the GNSS together with IMU high-frequency outputs to obtain very smooth positioning results [7,8]. When the GNSS signal is lost, the calculation errors in the integrated navigation system increase with IMU error accumulation.

Many researchers have studied single-IMU dead-reckoning navigation and optimized algorithms to reduce error accumulation in IMUs [4,9,10,11,12,13,14]. Low-pass filtering has been used to optimize the calculation results of low-cost shipboard IMUs [4]. However, the typical error in these systems remains over 100 m after 10 min. The influence of ship vibration, wind, and waves in the offshore environment causes significant noise in IMU attitude updating [11,12]. A “virtual vertical reference” method combined with a compass has been proposed to reduce IMU error divergence [13]. Nonetheless, under bad weather conditions, the angular error in roll and pitch measurements of low-cost IMUs can be up to 0.09°. Because the continuous and irregular navigation motion on the sea cannot be corrected by the device itself, low-cost IMUs alone are not reliable for navigation dead-reckoning [14].

Marine DVLs perform well in shallow water. Here, a four-beam DVL sonar, installed under the ship, measures the lateral and longitudinal velocities of the ship by transmitting and receiving sonar [15]. Only a few papers have discussed IMU/DVL coupling, which is used for autonomous underwater vehicle (AUV) navigation. AUV navigation is suitable for navigation along a predetermined fixed path [16]. However, to comply with local traffic center control and avoid collisions with other ships, vessels often cannot follow routing plans in the offshore area. Li et al. combined an IMU and 300 kHz DVL navigation using the least squares method [6]. By initial precise attitude alignment and ring-laser gyroscope, an experimental ship can sail 44 km in a river, with an error of 60 m.

In this study, we considered an IMU/DVL dead-reckoning navigation method for offshore vessels, in which significant wind and wave related noise in attitude estimates are expected. Two methods were applied and evaluated: the first was simple dead-reckoning using only the yaw rate of the IMU and DVL, while the second involved the integration of INS/DVL. In the case of simple dead-reckoning, some corrections were applied as follows. The DVL velocity was smoothed with IMU acceleration using a Kalman filter (KF), and the IMU directional accuracy was improved by dynamic zero velocity update (DZUPT). We estimated the bias of the yaw rate of the IMU as accurately as possible before the start of the GNSS outage. In addition, we considered the slip angle by using the yaw rate angle. For the integrated INS/DVL method, we used three gyroscopes and three accelerometers to monitor the attitude (roll, pitch, and yaw) of the ship, and hence improved the accuracy of position estimation. The gyrocompass on board the ship was used as a reference for the heading. To evaluate the methods, we obtained raw data including IMU, DVL, and GNSS from a 50 m training ship at Tokyo University of Marine Science and Technology called “Shiojimaru.” GNSS data were used as the reference points for performance evaluation. They were also used to estimate the IMU bias before the IMU/DVL dead-reckoning started. The difference in position accuracy between the two methods is evaluated and discussed. As for the reference flame, World Geodetic System (WGS)-84 was used for GNSS. Basically, we used Cartesian coordinates for position estimation using GNSS. In addition, ellipsoidal latitude, ellipsoidal longitude, and ellipsoidal height are also used in this paper, and are called geodetic coordinates. Both geodetic and Cartesian coordinates were mathematically related and converted. As for the local cartographic in horizontal plane, we set the reference positions deduced from cm-level RTK-GNSS results in geodetic coordinates and the deviations in horizontal plane were evaluated.

## 2. Method 1—Dead-Reckoning Navigation Using IMU and DVL

**Figure 1:**
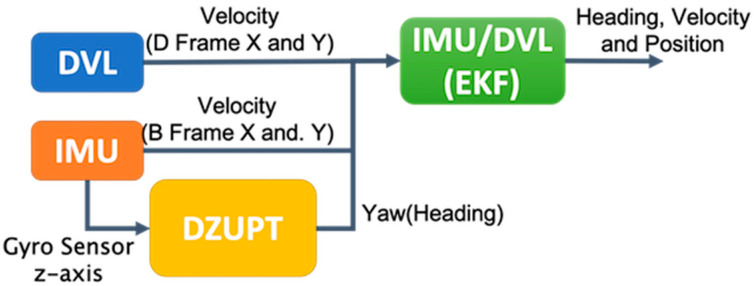
IMU and DVL integration architecture.

This section describes a method using an IMU and DVL to estimate velocity, direction, and position. For the IMU, only a yaw-rate gyroscope is used. GNSS data were used to estimate the direction before the GNSS outage started, as the IMU can detect changes in direction, but not direction itself. Figure 1 shows the algorithm flowchart. The level arm effect between the IMU and DVL is corrected for in the output of the DVL. While the real position of the DVL is at the bow of the bottom of the ship, the output is converted to the values for the center of the deck of the ship. The vertical position of the IMU is near the center of the deck. Since we primarily use the yaw-rate angle of the IMU for the heading, the level arm effects are typically small.

### 2.1. Direction Estimation

**Figure 2:**
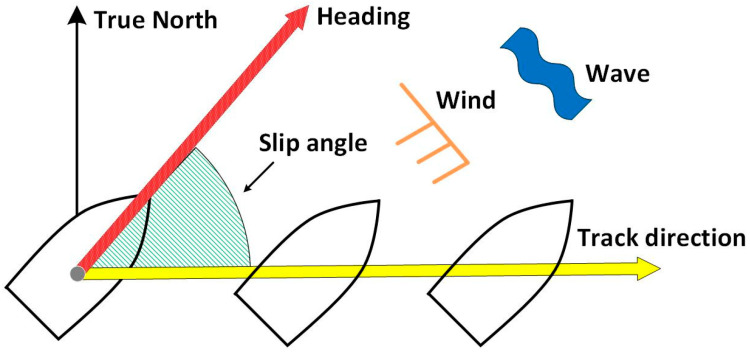
Heading and track directions.

In ship navigation, because of wind and currents, the ship heading direction is not always the same as the track direction (TD), as illustrated in Figure 2 [17]. An IMU gyroscope can provide the rate of change in direction of heading while the GNSS estimates direction from the velocity vector or position estimate, which is the TD. The angle between the heading and the TD is known as the slip angle and is influenced by many factors. A slip angle is frequently observed, especially when a ship is turning. In this study, the slip angle was estimated roughly using only the yaw-rate gyroscope of the IMU in the first test.

Here, we introduce two methods to estimate the direction. The first is the integration of the IMU with GNSS using a KF, described in Section 2.1.1. This is used primarily to correct the biases of the IMU. During a GNSS outage, the second method, using only the IMU, is used to estimate the direction. The direction can be estimated by accumulating the angular velocity in the IMU. However, it is necessary to correct the angular velocity bias because the IMU values drift easily. In addition, it is important to check and correct the drift of the IMU due to temperature changes. However, in the test conducted as part of this study, the temperature of the IMU varied by only 1°, and therefore the correction was not necessary.

#### 2.1.1. Direction Estimation by IMU with GNSS Using KF

As mentioned above, the direction of the ship can be determined by integrating the IMU with the GNSS. Normally, the GNSS direction is derived from the velocity deduced from the GNSS Doppler frequency or the GNSS carrier phase. The GNSS direction and the IMU angular velocity are integrated using the KF, during which two conditions of each output are satisfied simultaneously. The first condition is that the angular velocity of the IMU is within 0.0025 rad/s for 1 min. The second condition is that the standard deviation of the GNSS direction for 1 min be within 1°. The calculation formulas for the KF are given in Equations (1)–(11). Since we focused on the case of GNSS outage in this study, this integration was conducted only when the IMU and Doppler sonar dead-reckoning had started.

We used the GNSS based direction and velocity vector to initialize the yaw angle and accelerometers and, thus, set the initial alignment of the IMU. The accurate GNSS-based direction and velocity vectors were also used to predict biases in the IMU. The estimated directional and velocity accuracies are within 1° and 1 cm/s, respectively.

(1) $$x_{k_{dir}}=\left[\begin{array}{cc} \theta_{G_{k}{}_{dir}}, & \omega_{G_{k}{}_{dir}} \end{array}\right]\;\;\;\;\;\;\;\Phi_{dir}=\left[\begin{array}{cc} 1 & \Delta t\\ 0 & 1 \end{array}\right]\;\;R_{dir}=\left[\begin{array}{cc} \sigma_{\theta_{G}}^{2} & 0\\ 0 & \sigma_{\omega_{z}}^{2} \end{array}\right]$$

$\theta_{G_{k}}$: GNSS direction [°]
$\omega_{G_{k}}$: Gyroscope angular velocity with bias taken into account as shown in Equation (14) [°/s]
*dir*: Indicates that dir is a calculation of direction.
Δt = 0.2 [*s*]…GNSS frequency;
$\sigma_{\theta_{G}}=1.0\left[{}^{\circ}\right]$…GNSS error standard deviation of orientation;
$\sigma_{\omega_{z}}=0.02\;\left[{}^{\circ}/s\right]$…IMU error standard deviation of angular velocity.
$x_{k_{dir}}$: State vectors
$\Phi_{dir}$: State − space matrix
$R_{dir}$: Covariance matrix
$k_{dir}$: Number of updates of KF for direction estimation

The system noise matrix Q:

(2) $$Q_{k_{dir}}=\left[\begin{array}{cc} 10^{-4} & 0\\ 0 & 10^{-4} \end{array}\right]$$

The process noise G:

(3) $$G_{dir}=\left[\begin{array}{cc} 1 & 0\\ 0 & 1 \end{array}\right]$$

The measurement matrix H:

(4) $$H_{dir}=\left[\begin{array}{cc} 1 & 0\\ 0 & 1 \end{array}\right]$$

The discrete state-space model:

(5) $$x_{k_{dir}+1}=\Phi_{dir}x_{k_{dir}}+Gw_{k}$$

Observation equation:

(6) $$y_{k_{dir}}=H_{k_{dir}}x_{k_{dir}}+v_{k}$$

Correction of the current state from the estimate before the first step:

(7) $${\hat{x}}_{k_{dir}|k_{dir}}={\hat{x}}_{k_{dir}|k_{dir}-1}+K_{k}\left(y_{k}-H_{k_{dir}}{\hat{x}}_{k|k-1}\right)$$

Estimation of the value of the next step:

(8) $${\hat{x}}_{k_{dir}+1|k_{dir}}=F_{k_{dir}}{\hat{x}}_{k_{dir}|k_{dir}}$$

Kalman gain update:

(9) $$K_{k_{dir}}=P_{k_{dir}|k_{dir}-1}H_{k_{dir}}^{T}{\left(H_{k_{dir}}P_{k_{dir}|k_{dir}-1}H_{k_{dir}}^{T}+R_{k_{dir}}\right)}^{-1}$$

Update the error covariance matrix $P_{k_{dir}|k_{dir}}$:

(10) $$P_{k_{dir}|k_{dir}}=P_{k_{dir}|k_{dir}-1}-K_{k_{dir}}H_{k_{dir}}P_{k_{dir}|k_{dir}-1}$$

Error covariance matrix estimation:

(11) $$P_{k_{dir}+1|k_{dir}}=F_{k_{dir}}P_{k_{dir}|k_{dir}}F_{k_{dir}}^{T}+G_{k_{dir}}Q_{k_{dir}}G_{k_{dir}}^{T}$$

Based on the results of several experiments, the initial value of $P_{k_{dir}|k_{dir}}$ was set to diag([10 10]). The value of the error covariance, R, of the KF was determined as follows: GNSS direction is known to contain a noise-like error of approximately ±1.0° under normal navigation speed. Therefore, we set $\sigma_{\theta_{G}}=1.0{}^{\circ}$. To calculate the error standard deviation of the angular velocity, the standard deviation $\sigma_{\omega_{z}}=0.02$ of the output value (noise) at rest was acquired before the experiment was set. This output of the filtered direction was then as the initial direction for dead-reckoning in the following test. Furthermore, the bias of the yaw-rate angle was estimated using this method, and the calculated yaw-rate angle bias was also used as the initial value of the following test.

#### 2.1.2. Direction Estimation Using Only IMU

Here, the direction was estimated at 100 Hz using only the Z-axis angular velocity. When calculating the direction with the IMU, it is necessary to obtain the initial direction from another sensor. In this experiment, the initial direction was obtained from the last GNSS epoch:

(12) $$\theta_{t}=\theta_{t-1}+\omega_{G_{k}{}_{dir\left(t-1\right)}}\Delta t$$

$$\Delta t=0.01\left[s\right],\;\omega_{G_{k}{}_{dir\left(t-1\right)}}=\;\omega_{G_{k}{}_{dir}}\;\mathrm{at}\;\mathrm{time}\;t-1\;\left[{}^{\circ}/s\right],\;\theta =\mathrm{Azimuth}\;\left[{}^{\circ}\right]$$

Normally, the angular velocity of an IMU has a bias error owing to the temperature change of the sensor and the elapsed time. The estimated bias, determined using the GNSS and IMU, was removed as an initial bias. To cater for subsequent bias changes, the DZUPT (dynamic zero velocity update) process was used. In DZUPT, the average of the sensor output value is calculated when the sensor is stationary, and the zero-point is updated using the average value as a bias. However, there is some movement on the ship at all times, even when anchored; and therefore the sensor will never be completely stationary.

As the angular velocity around the Z-axis does not change while going straight, the average value of the angular velocity while going straight can be estimated and removed. The average value of the angular velocity in the experimental section, 0.0025 rad/s, was used as the threshold value to determine whether the vehicle is going straight. By subtracting the average value in Equation (13) of the angular velocity for 60 s (6000 epochs in 100 Hz) when going straight, as shown in Equation (14), the bias due to the time change was removed [11].

$b_{\omega }{}_{t}$: Bias estimated using DZUPT:

(13) $$b_{\omega }{}_{t}=\frac{1}{6000}\overset{6000}{\underset{t=1}{\sum }}\omega_{t}$$

(14) $$\omega_{G_{k}{}_{dir}}=\left(\omega_{t}-b_{\omega }{}_{t}\right)$$

$$\omega_{t}=\;Z-axis\;angular\;velocity\;at\;time\;t\;\left[{}^{\circ}/s\right]$$

### 2.2. Velocity Estimation

For estimating the velocity, we considered the speed information included in the NMEA output from the GNSS receiver, the ground speed by DVL, and the IMU acceleration integral value.

#### 2.2.1. Velocity Observation by GNSS

Velocity information deduced from GNSS can be used until GNSS failure. A Doppler shift occurs in the GNSS signal due to the movement of the satellite and receiving antenna. Since the moving speed on the satellite side is accurately known from the satellite orbit information ephemeris included in the GNSS signal, the speed and direction on the receiver side can be obtained from the difference between the relative speed obtained from the Doppler shift and the speed of the satellite. The accuracy of the velocity vector from the GNSS is generally within 1 cm/s under open-sky conditions at sea.

#### 2.2.2. Velocity Observation with DVL

The DVL obtains ground speed using the Doppler shift generated by the movement of the ship between transmitted and received sound waves. Hereinafter, the speed obtained by the DVL is referred to as the sonar speed. The DVL mounted on the ship shows only the X-axis velocity ($V_{x}$) in the bow direction and the Y-axis velocity ($V_{y}$) perpendicular to it on the display of the bridge, and there is no system to store the data. Therefore, the speed shown on the screen was visually confirmed and converted into data. The final horizontal velocity was calculated using Equation (15).

(15) $$V=\sqrt{V_{x_{k}}^{2}+V_{y_{k}}^{2}}$$

$V_{x_{k}}$: Sonar speed in X axis [m/s]
$V_{y_{k}}$: Sonar speed in Y axis [m/s]

#### 2.2.3. Speed Estimation by KF

The speed data from the DVL includes sudden water flow noise. In addition, there is a problem with the IMU speed drifts. Therefore, the velocities from both sensors were combined using the KF Equations (8)–(14) used in Section 2.1.2. The observed values x, the linear model $F_{speed}$, and the error covariance $R_{speed}$ were calculated using Equations (16) and (17). The error information from the DVL was confirmed in the catalog, as shown in Section 4 later, but since the information is very old, an error standard deviation of 0.11 m/s, when compared with GNSS, was used. As the voyage speed during this experiment was about 9 knots, the measurement error of 2% was about 0.09 m/s. This was judged a reasonable error value when compared with the catalog value. In addition, considering the sway of the vessel, the standard deviation of the acceleration error was set to 0.06 in stationary state.

(16) $$x_{k_{speed}}=\left[\begin{array}{cccc} V_{x_{k}{}_{speed}} & V_{y_{k}{}_{speed}} & a_{x_{k}{}_{speed}} & a_{y_{k}{}_{speed}} \end{array}\right]\;$$

(17) $$F_{speed}=\left[\begin{array}{cccc} 1 & 0 & \Delta t_{S} & 0\\ 0 & 1 & 0 & \Delta t_{S}\\ 0 & 0 & 1 & 0\\ 0 & 0 & 0 & 1 \end{array}\right]\;R_{speed}=\left[\begin{array}{cccc} \sigma_{V_{x}}^{2} & 0 & 0 & 0\\ 0 & \sigma_{V_{y}}^{2} & 0 & 0\\ 0 & 0 & \;\sigma_{a_{x}}^{2} & 0\\ 0 & 0 & 0 & \;\sigma_{a_{y}}^{2} \end{array}\right]$$

$\Delta t_{S}$ = 1 [s]…DVL observation period
$\sigma_{V_{x}}$ = 0.11 [m/s]…DVL speed error Standard deviation in X direction
$\sigma_{V_{y}}$ = 0.11 [m/s]…DVL speed error Standard deviation in Y direction
$\sigma_{a_{x}}\;$= 0.06 [m/s^2^]…IMU acceleration error standard deviation in X direction
$\sigma_{a_{y}}$ = 0.06 [m/s^2^]…IMU acceleration error standard deviation in Y direction
$k_{speed}$: Number of updates of KF for velocity estimation

In the equations above, the matrix $R_{speed}$ is the DVL speed error covariance, which is the standard deviation of the difference between the DVL and the GNSS velocity. The IMU acceleration is obtained in advance for approximately 1 h in a stationary state; and the standard deviation is used as the error standard deviation of the IMU.

### 2.3. Position Estimation

The horizontal position was evaluated in this study because the ship was always on the sea. In order to simplify the calculation, we did not consider the roll and pitch of the ship, and only used yaw changes for dead-reckoning. The current position was estimated by integrating the azimuth angle obtained from the IMU and the moving distance obtained from the velocity from Equations (18) and (19). The reference positions for evaluating the temporal horizontal errors were deduced from the real-time kinematic (RTK)-GNSS positions of the target antenna installed on the ship.

(18) $$px_{n}=px_{n-1}+V\cdot \sin\left(\theta_{t}\frac{\pi }{180}\right)\cdot \Delta t$$

(19) $$py_{n}=py_{n-1}+V\cdot \cos\left(\theta_{t}\frac{\pi }{180}\right)\cdot \Delta t$$

where px, py = XY coordinates when the reference station is 0 m, and V = velocity [m/s] Δt = 1 or 0.2 (velocity interval).

## 3. Method 2—INS/DVL Integrated Navigation

**Figure 3:**
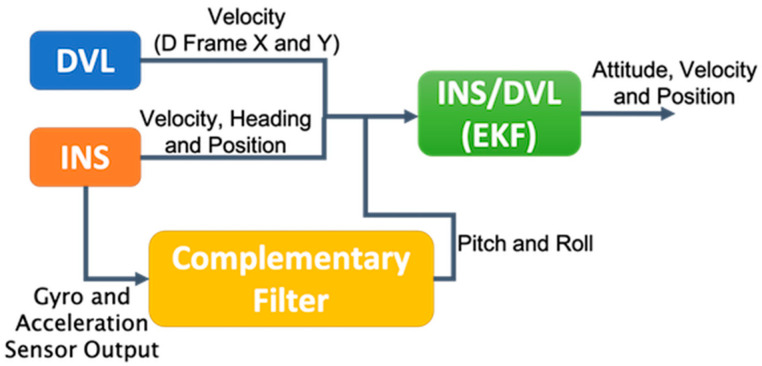
Loosely coupled INS/DVL EKF integration architecture.

This section describes the method of using an INS and DVL loosely coupled extended KF (EKF) integration system. The sensors for reference are different from those described in Section 2. GNSS data were used as a reference to evaluate each error in Section 2 and Section 4. On the other hand, GNSS data, a gyrocompass, and a FOG were used to evaluate each error in Section 3 and Section 4. In this study, we developed an EKF based on NaveGo [13,18,19,20]. For the inertial navigation calculation, the algorithm described in Chapter 17 of [21] was used. The MATLAB complementary Filter System object was used for calculating roll and pitch using accelerometer and gyroscope sensor data, with an accelerometer gain of 0.01 [22]. The loosely coupled INS/DVL integration architecture is shown in Figure 3.

### 3.1. Parameter Estimation by Allan Variance

**Figure 4:**
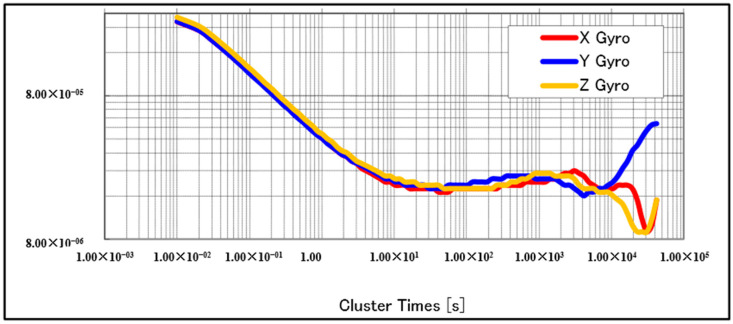
Allan variance (AV) plots for gyroscopes.

**Figure 5:**
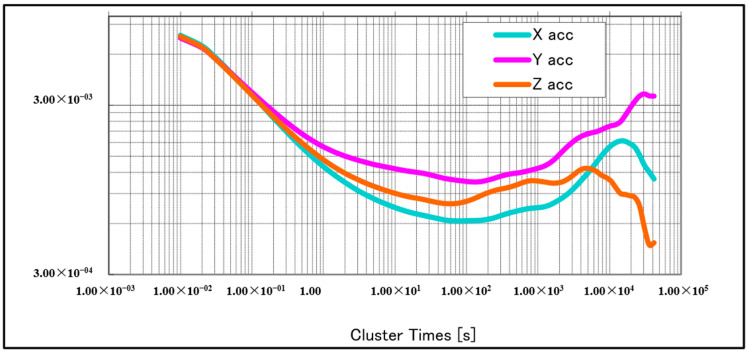
AV plots for accelerometers.

We used a commercial CSM-MG100 IMU device for these tests. As the CSM-MG100 is already packaged, and the details of the sensors used are unknown, we estimated the accuracy of the sensor using Allan variance (AV) analysis. Moreover, a more detailed profile from a specific unit is required to later use the level of the sensor noise to configure a KF, which will be part of an integrated navigation system (INS/DVL) [12]. Static bias, standard deviation (STD), angle velocity random walk, and bias instability are shown in Table 1. Figure 4 and Figure 5 show the AV plots from the gyroscopes and accelerometers, respectively.

### 3.2. INS/DVL Integration

The error equation for the direction cosine matrix relating coordinate Frames B and L($C_{B}^{L}$) is derived in the navigation coordinate frame and the local-level coordinate frame (wander-azimuth) as follows [23,24]:

(20) $$\dot{{\underline{\gamma }}^{L}}=-\left(\underline{\omega }{}_{\mathrm{IL}}^{L}\times \right){\underline{\gamma }}^{L}-C_{B}^{L}\delta \tilde{\underline{\omega }}{}_{\mathrm{IB}}^{B}+\delta \underline{\omega }{}_{\mathrm{IL}}^{L}+C_{N}^{L}\frac{1}{R}\left(\underline{u}{}_{\mathrm{ZN}}^{N}\times {\delta \underline{v}}^{N}\right)$$

(21) $$\delta C_{B}^{L}=-{\underline{\gamma }}^{L}\times C_{B}^{L}$$

(22) $$\delta \overset{˜}{\underset{\_}{\omega }}{}_{\mathrm{IB}}^{B}=\eta_{g}+b_{g}+\eta_{g\delta b}$$

where $\eta_{g}$, $b_{g}$ and $\eta_{g\delta b}$ are an angle random walk noise, static bias and discrete sequence related to bias instability $\delta {\underline{b}}_{g}$, respectively [18].

The error equation for the ${\delta \underline{v}}^{L}$ is derived while ignoring errors in the Coriolis terms and the gravity vector [25], but taking into account ${\delta \underline{v}}^{N}$, which is the error in velocity relative to the earth measured in the N Frame [24] part:

(23) $$\delta {\overset{˙}{\underset{\_}{v}}}^{L}=\left(C_{B}^{L}\underline{a}{}_{\mathrm{SF}}^{B}\right)\times {\underline{\gamma }}^{L}+C_{B}^{L}\delta \overset{˜}{\underset{\_}{a}}{}_{\mathrm{SF}}^{B}-C_{N}^{L}\left(2\underline{\omega }{}_{\mathrm{IE}}^{N}+\underline{\omega }{}_{\mathrm{EN}}^{N}\right)\times {\delta \underline{v}}^{N}$$

(24) $$\delta \overset{˜}{\underset{\_}{a}}{}_{\mathrm{SF}}^{B}=\eta_{f}+b_{f}+\eta_{f\delta b}$$

where, $\eta_{f}$, $b_{f}$ and $\eta_{f\delta b}$ are a velocity random walk noise, static bias and discrete sequence related to bias instability $\delta {\underline{b}}_{f}$, respectively [18]. The $C_{N\;}^{L}$ is the direction cosine matrix that transforms vectors from N to L-Frame [24] defined as:

(25) $$C_{N}^{L}=\left[\begin{array}{ccc} 0 & 1 & 0\\ 1 & 0 & 0\\ 0 & 0 & -1 \end{array}\right]$$

${\underline{\gamma }}^{L}$ is the small-angle rotation vector error associated with $\delta C_{B}^{L}$. $C_{B}^{L}$ is the direction cosine matrix that transforms a vector from its B-frame projection form to its L-frame projection form. ${\underline{a}}_{\mathrm{SF}}^{B}$ is the specific force acceleration vector in the B Frame [24]. $\delta {\underline{\tilde{\omega }}}_{\mathrm{IB}}^{B}$ and $\delta {\overset{˜}{\underset{\_}{a}}}_{\mathrm{SF}}^{B}$ are the gyroscope sensor and acceleration sensor measurement errors, respectively. ${\underline{\omega }}_{\mathrm{IL}}^{L}$, $\underline{\omega }{}_{\mathrm{IE}}^{N}$, and $\underline{\omega }{}_{\mathrm{EN}}^{N}$ are the angular rates of the local-level coordinate frame to the inertial frame, the angular rate of the earth frame relative to the inertial frame, and the angular rate of the navigation frame to the earth frame, respectively. $\delta \underline{\omega }{}_{\mathrm{IL}}^{L}$ is the error in $\underline{\omega }{}_{\mathrm{IL}}^{L}$, and $\underline{u}{}_{\mathrm{ZN}}^{N}$ is the unit vector relative to the earth in N-Frame axes [24]. The INS error state vectors are summarized below:

(26) δx_^=[γ_L,δv_L,δb_g,δb_f]T

where $\delta {\underline{b}}_{g}$ and $\delta {\underline{b}}_{f}$ are the bias instability estimation vectors for the gyroscope and accelerometer, respectively. Similar to NaveGo [20], the continuous state-space model of the system and the discrete state-space model of the system are used as follows [26]:

(27) $$\delta {\dot{\hat{x}}}_{\left(t\right)}=F_{\left(t\right)}{\delta \hat{x}}_{\left(t\right)}+G_{\left(t\right)}u_{\left(t\right)}+\zeta_{\left(t\right)}$$

(28) $$\delta {\hat{y}}_{\left(t\right)}=H\delta {\hat{x}}_{\left(t\right)}+v_{\left(t\right)}$$

(29) $$\delta {\hat{x}}_{\left(+\right)}=\Phi \delta \hat{x}+Gu+\zeta$$

(30) $$\delta \hat{y}=H\delta \hat{x}+v$$

Vectors ζ and v are known as the driving noise and measurement noise with zero-mean Gaussian white noise, respectively [20]. The state-space matrices are:

(31) $$F_{\left(t\right)\left\{12\times 12\right\}}=\left[\begin{array}{cccc} -\left(\underline{\omega }{}_{\mathrm{IL}}^{L}\times \right) & -\frac{1}{R}C_{N}^{L}\left(\underline{u}{}_{\mathrm{ZN}}^{N}\times \right) & -{\hat{C}}_{B}^{L} & 0\\ {\hat{C}}_{B}^{L}{\underline{a}}_{\mathrm{SF}}^{B}\times & 0 & 0 & \hat{C}{}_{B}^{L}\\ 0 & 0 & -\frac{1}{\tau_{g}} & 0\\ 0 & 0 & 0 & -\frac{1}{\tau_{f}} \end{array}\right]$$

(32) $$\hat{C}{}_{B}^{L}=\left(I_{3}-\Gamma^{L}\right)C_{B}^{L},$$

where $\underline{u}{}_{\mathrm{ZN}}^{N}$ is a unit vector relative to the earth in the N-frame axes [24]. $\tau_{g}$ and $\tau_{f}$ are the correlation times of the dynamic accelerometer and gyroscope biases, respectively [27]. $\hat{C}{}_{B}^{L}$ is the direction cosine matrix with an error. $\Gamma^{L}$ is a skew-symmetric operator associated with ${\underline{\gamma }}^{L}$. The vector u is defined as:

(33) $$u={\left[{\underline{\tilde{\omega }}}_{\mathrm{IB}}^{B},\underline{\tilde{a}}{}_{\mathrm{SF}}^{B},{\underline{\eta }}_{g\delta b},{\underline{\eta }}_{f\delta b}\right]}^{T}$$

(34) $$\underline{\tilde{\omega }}{}_{\mathrm{IB}}^{B}=\underline{\omega }{}_{\mathrm{IB}}^{B}+\delta \underline{\tilde{\omega }}{}_{\mathrm{IB}}^{B}$$

(35) $$\;\underline{\tilde{a}}{}_{\mathrm{SF}}^{B}={\underline{a}}_{\mathrm{SF}}^{B}+\delta \underline{\tilde{a}}{}_{\mathrm{SF}}^{B},$$

where ${\underline{\eta }}_{g\delta b}$ and ${\underline{\eta }}_{f\delta b}$ are obtained in an iterative fashion with ${\underline{\eta }}_{g\delta b\left(1\right)}={\underline{b}}_{g}$ and ${\underline{\eta }}_{f\delta b\left(1\right)}={\underline{b}}_{f}$. ${\underline{b}}_{g}$ and ${\underline{b}}_{f}$ are static bias varies [20].

The propagation interval is 1 s, and the IMU system noise matrix *Q* is defined as [27]:

(36) $$Q=\left[\begin{array}{cccc} Q_{11} & Q_{21}^{T} & -\frac{1}{2}S_{bgd}\tau_{s}^{2}\hat{C}{}_{B}^{L} & 0_{3}\\ Q_{21} & Q_{22} & -\frac{1}{3}S_{bgd}\tau_{s}^{3}F_{21}\hat{C}{}_{B}^{L} & \frac{1}{2}S_{bad}\tau_{s}^{2}\hat{C}{}_{B}^{L}\\ \frac{1}{2}S_{bgd}\tau_{s}^{2}\hat{C}{}_{B}^{L} & \frac{1}{3}S_{bgd}\tau_{s}^{3}F_{21}\hat{C}{}_{B}^{L} & S_{bgd}\tau_{s}I_{3} & 0_{3}\\ 0_{3} & \frac{1}{2}S_{bad}\tau_{s}^{2}\hat{C}{}_{B}^{L} & 0_{3} & S_{bad}\tau_{s}I_{3} \end{array}\right]$$

where $S_{bad}$, and $S_{bgd}$ are accelerometer bias variation and gyroscope bias variation, respectively [27]. Submatrices $Q_{xx}$ are shown in Equation (14.81) of [27]. $I_{3}$ and $0_{3}$ are the 3 × 3 identity matrix and zero matrix, respectively. $F_{xy}$ is value of the row x and column y of F in Equation (31). The covariance matrix *R* is defined using the DVL STD as:

(37) $$R_{\left\{3\times 3\right\}}=\left[\begin{array}{ccc} \sigma_{V_{x}}{}^{2} & 0 & 0\\ 0 & \sigma_{V_{y}}{}^{2} & 0\\ 0 & 0 & 1 \end{array}\right]$$

The third row is retained only for the calculation and future use, and is not used for any result. The DVL measurements in the local frame are calculated as:

(38) $$\hat{\underline{v}}{}_{\mathrm{DVL}}^{L}=\hat{C}{}_{B}^{L}{\left(C_{B}^{D}\right)}^{T}\hat{\underline{v}}{}_{\mathrm{DVL}}^{D}$$

$C_{B}^{D}$ is the direction cosine matrix used as a misalignment matrix between the IMU and DVL. After several experiments, we set $C_{B}^{D}$ as $\phi_{BD}=0{}^{\circ},\;\theta_{BD}=0{}^{\circ},\;\psi_{BD}=2.6{}^{\circ}$. The scale factor error [6] was not included in this study. The measurement model of the KF is as follows [6]:

(39) $$\delta \hat{y}=\hat{\underline{v}}{}_{\mathrm{INS}}^{L}-\hat{\underline{v}}{}_{\mathrm{DVL}}^{L}=\delta \underline{v}{}_{\mathrm{INS}}^{L}-\left[\left(C_{B}^{L}{\left(C_{B}^{D}\right)}^{T}\underline{v}{}_{\mathrm{DVL}}^{D}\right)\times \right]{\underline{\gamma }}^{L}$$

(40) $$H=\left[-C_{B}^{L}{\left(C_{B}^{D}\right)}^{T}\underline{V}{}_{\mathrm{DVL}}^{D}{\;I}_{3}\;0_{3}\right]$$

$\hat{\underline{v}}{}_{\mathrm{INS}}^{L}$ is the INS velocity output in the L Frame with errors. $\hat{\underline{v}}{}_{\mathrm{DVL}}^{L}$ is the DVL velocity in the L Frame with errors.

The EKF is updated in the same manner as NaveGo [18,19,20] with DVL update time as shown Equation (41). The numerical values of the parameters used in this experiment are listed in Table 2.

(41) Φ=I+FΔt

Δt: DVL sampling rate with 1 [s]

## 4. Experiment and Results

### 4.1. Experiment Outline

To test the algorithms described in Section 2 and Section 3, an experiment was designed as follows. The experiment assumed that while sailing in Tokyo Bay, interference was detected near Tokyo Haneda Airport, and the GNSS direction before the interference was set as the initial direction of the IMU direction. Subsequently, the IMU azimuth, DVL velocity, and IMU acceleration with the KF were compared with GNSS-derived velocity/direction information and RTK-GNSS positions to evaluate the accuracy. The data were obtained by installing an IMU and GNSS receiver on the “Shiojimaru.” In addition, the DVL output of the ship was recorded.

Table 3 summarizes the sensor information used in this experiment. The IMU was the CSG-MG100, manufactured by Tokyo Aircraft Instrument Co., Ltd, Tokyo, Japan. and equipped with a time-synchronization function with GNSS. This IMU can detect three-axis acceleration and three-axis angular velocity. During the experiment, the IMU was installed with the bow direction along the X-axis, the starboard direction perpendicular to it as the Y-axis, and the vertically downward direction as the Z-axis. The four-beam DVL ATLAS DOLOG SYSTEM was installed on the bottom of the ship in front of the vessel bow thruster. In the fore-aft and lateral directions, the DVL can obtain ground speeds for up to 200 m depths. For this experiment, we set the DVL error at 0.2% of the measured value.

Since multiple KFs were used to integrate these sensors, we will introduce them with the results. The results of the RTK positioning between the Trimble Net R9 Marine Network Reference Station and the Trimble SPS855 receiver installed on the vessel were used for comparing the estimated directions, speeds, and positions. In this experiment, after the ship departed from the port, it used GNSS to sail normally, and carried out dead-reckoning experiments when it sailed to the waters of Haneda International Airport. The ship obtained GNSS, IMU, and DVL data through tests in open waters at a constant speed. In order to maximize the errors, the ship was maneuvered as vigorously as possible. After several straight and continuous large-angle turns, the difference between the heading and TD continued to change, and the dead-reckoning error was affected by its own inertia, water current, and wind. Using GNSS data as a reference value, the error of dead-reckoning was obtained.

### 4.2. Results with First Method

**Figure 6:**
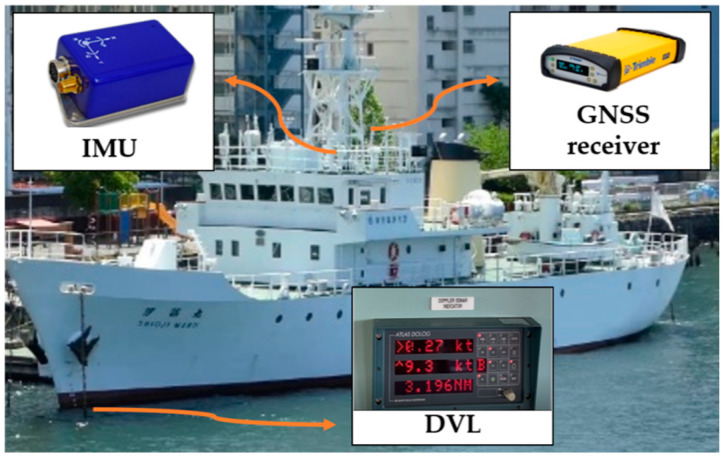
Instrument setup.

**Figure 7:**
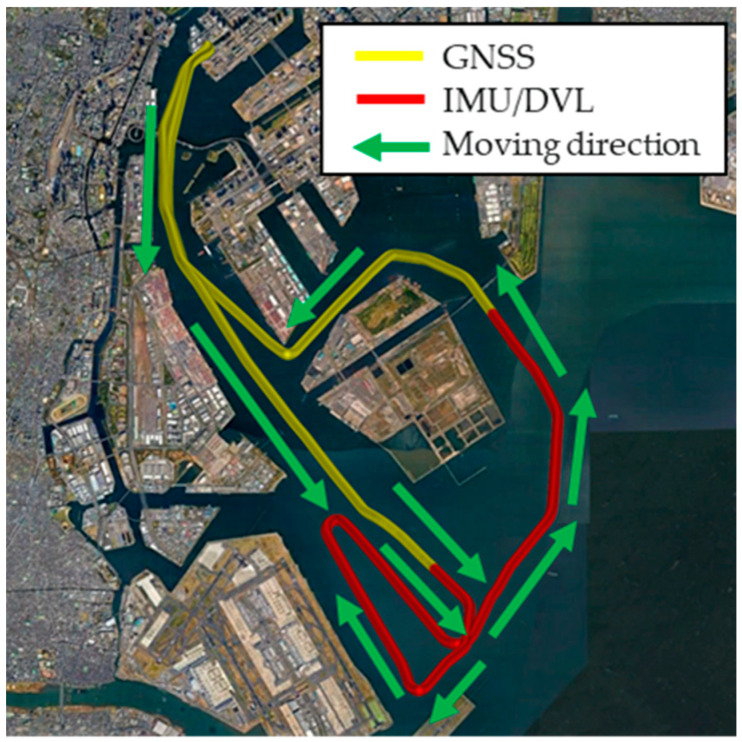
Voyage tracking using Google Earth.

In this experiment, we obtained a 1 h voyage dataset of combined IMU/DVL and GNSS tracking. Figure 6 shows the sensors used in this experiment. Figure 7 shows that the vessel first followed the yellow line. In the red-line area, the vessel navigated by the IMU/DVL dead-reckoning. The vessel made some large-angle turns and then went straight and turned left twice. The results are discussed in the following.

#### 4.2.1. Evaluation of Estimated Direction

**Figure 8:**
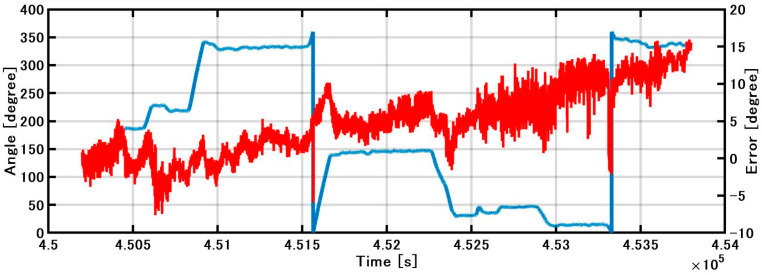
Estimated direction and error.

In Figure 8, the blue line shows the difference between the IMU direction and the GNSS true direction. The red line shows the direction obtained by the KF based on the gyroscope. A large difference occurs when the heading changes and the maximum error accumulates to 15 degrees in 1 h. Even if the zero points are updated by DZUPT when going straight, the bias is constantly changing, so an error gradually occurs between the update and the next update. In addition, the bias cannot be completely removed by DZUPT processing even when going straight due to the wind and waves, and the zero-point shifts slightly to the left or right at each epoch update. Consequently, when the angular velocity with the zero points slightly deviated is integrated to obtain the direction, the shape swings to the left or right. Furthermore, the effect of the slip angle cannot be corrected, as there is no GNSS.

We tested a simple case of slip-angle correction when the ship changed her course largely. There were seven course changes during the 1 h test. We noted that the temporal yaw-rate angle followed a similar trend to the temporal-slip angle between the heading and TD. Therefore, we used this value of yaw-rate angle as the slip angle and multiplied it by a factor to match the size of slip angle. In fact, the maximum slip angle based on the post-processed data was about 7° in the case of a 180° turn during the test. We decided the factor based on this maximum value. In the following test section, the results with slip-angle correction are compared with the results without slip-angle correction.

#### 4.2.2. Evaluation of Speed Estimation

**Figure 9:**
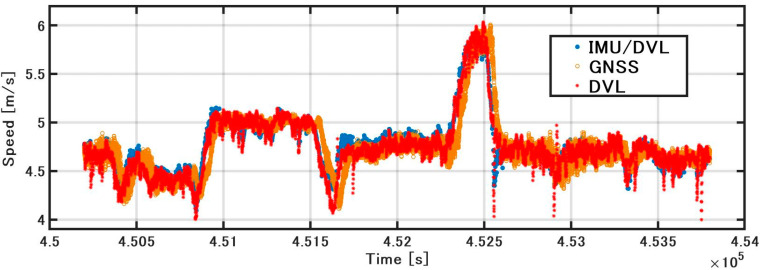
Speed comparison.

**Figure 10:**
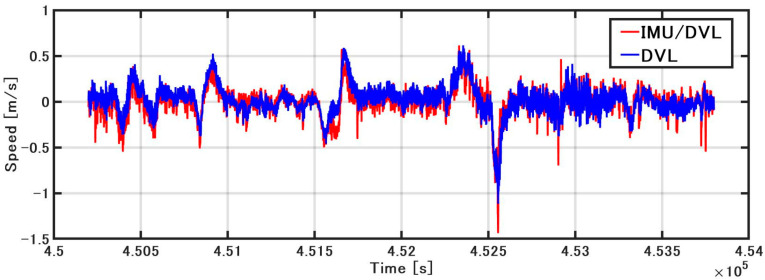
Speed error comparison.

As shown in Figure 9, when the GNSS velocity, the speed obtained by integrating the acceleration of the IMU (hereinafter referred to as the IMU speed), and the DVL speed are compared, the accuracy of the IMU speed deteriorates with the increase in bias. Because the acceleration includes gravitational acceleration, it is difficult to estimate the bias with DZUPT, due to the vertical movement of the vessel. Even if DZUPT can be applied, the instability errors of the bias and velocity cannot be fully removed. DVL and GNSS velocity values are generally equal, but the speed of DVL includes some jumps. These jump values appear as large error factors in position estimation. The cause of the jump is generally considered to be the effect of hull sway and air bubbles, but the specific cause is unknown. To confirm this error in detail, as shown in Figure 10, the error was calculated by subtracting the GNSS speed (RMC output from NMEA) from the sonar speed. The STD was 0.11 m/s, and the maximum error was about 0.8 m/s. As a result of constructing a speed estimation KF using this STD, the noise of the DVL is generally smoothed; however, the accuracy is lower than that of GNSS or DVL alone at approximately 452,500 s. The STD of the error in IMU/DVL was 0.09 m/s, and the maximum error was 0.53 m/s, which was better than the result of the DVL alone. 

#### 4.2.3. Position Estimation Result

**Figure 11:**
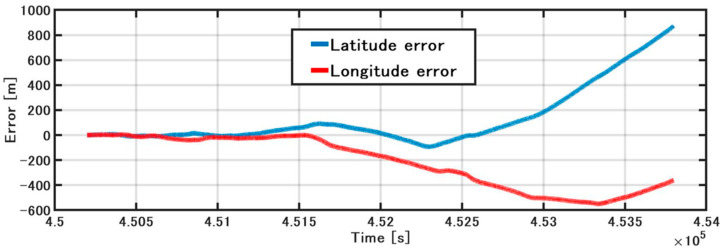
IMU/DVL position error.

Figure 11 shows the position error of the IMU/DVL dead-reckoning. The attitude error changed slightly during ship turning and increased at the point when the vessel finished the turn and went straight, with a final latitude error of 873 m. Initially, the longitude error increased slowly and finally reached −357 m. Due to the ship-steering operation, the vessel had a different force and error model at the different heading. This is why the latitude and longitude errors sometimes increased and sometimes decreased. In addition, we investigated the position error with rough slip-angle correction for large turns. The final latitude error was reduced from 873 m to 851 m, and the final longitude error was reduced from −357 m to −314 m. These results indicate a need for further investigation of slip-angle errors in the future. 

### 4.3. Results with Second Method

For INS/DVL, the initial conditions, such as velocity, attitude, and position, were estimated using INS/GPS. The same data were used in Section 4.2. As the attitude was not estimated at the starting point, we input the attitude estimated by INS/GPS as a constant value before starting the estimation. To obtain reference values for the attitude of the B-frame, the gyrocompass installed on the vessel was used for the heading, and a JCS7402-A [28] FOG was used for the roll-angle and pitch-angle reference values. The specifications of each of these devices are listed in Table 3 and Table 4. FOG specifications are shown in Table 5. When the true value is unknown or the error is expected to be included in the reference value, it is indicated as a “difference” rather than an “error.”

#### 4.3.1. Evaluation of Estimated Attitude

**Figure 12:**
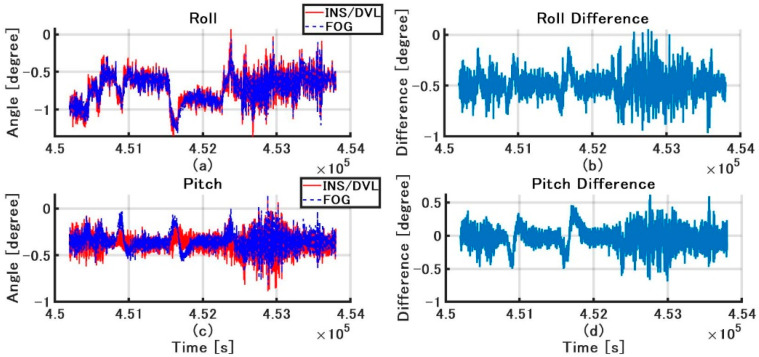
(**a**) Roll angle of INS/DVL and FOG; (**b**) Roll difference between INS/DVL and FOG; (**c**) Pitch angle of INS/DVL and FOG; (**d**) Pitch difference between INS/DVL and FOG.

**Figure 13:**
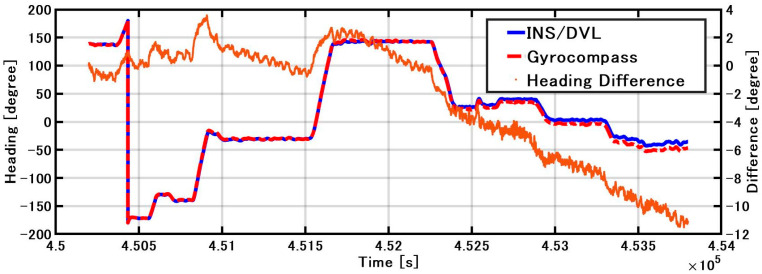
INS/DVL and gyrocompass heading and difference.

The roll and pitch outputs of INS/DVL and FOG and the differences in each output between them are shown in Figure 12. The difference between the FOG and INS/DVL pitch angle was 0.62° to −0.69°. By contrast, the difference in roll angle between the INS/DVL and FOG was 1.1° to −0.9°. Figure 12c shows that the INS/DVL had a poor response to small angle variations. We found that the drift in roll and pitch can be suppressed using a complementary filter for the sea conditions experienced during this experiment. The heading output of the INS/DVL and gyrocompass and the differences between them are shown in Figure 13. The heading error increased throughout the experiment to a maximum of −11.10°, which is a factor that affected the final position error described below. The heading error did not increase initially until about 4.515 × 10^5^ s. We think this is because the bias was removed by turning nearly 360°. Figure 13 shows that the difference increased exponentially when turning, and increased at a constant rate when going straight. For this difference, we can create a detailed sensor-error model, estimating the error when GNSS is available, performing the same process as DZUPT when the vessel is moving straight ahead, or estimating the error using the gyrocompass. In the future, we intend to study a method of bias estimation according to the available equipment. 

#### 4.3.2. Evaluation of Speed Estimation

**Figure 14:**
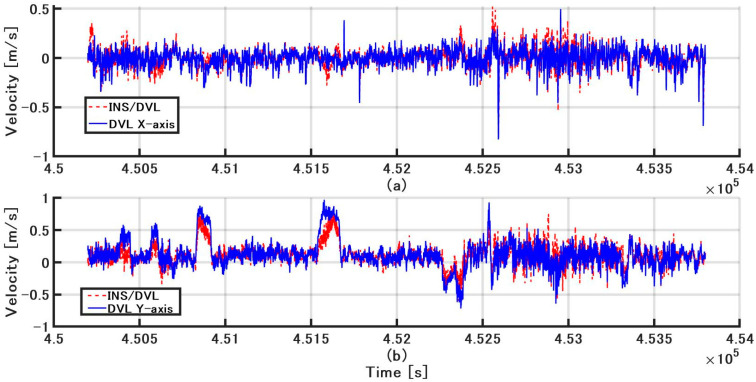
(**a**) INS/DVL and DVL x-axis velocities minus estimated value, respectively; (**b**) INS/DVL and DVL y-axis velocities minus estimated value, respectively.

The DVL and INS/DVL speed are compared in the B-frame. The estimated value is the value calculated from the attitude from the FOG and gyrocompass and the velocity determined by GPS using a trajectory generator [21]. The difference between the INS/DVL and estimated values for the B-frame X-axis and Y-axis are shown in Figure 14a,b, respectively. The mean difference in the X-axis velocity of INS/DVL and sonar velocity against the estimated value were −1.58 × 10^−4^ (STD of 9.322 × 10^−2^) and −1.53 × 10^−4^ (STD of 9.631 × 10^−2^), respectively. The average difference between the Y-axis INS/DVL and sonar velocities and the estimated values were 1.15 × 10^−1^ (STD of 1.580 × 10^−1^) and 1.21 × 10^−1^ (STD of 2.168 × 10^−1^), respectively, which were larger than that of the X-axis. As can be seen from the average, a bias-like component was detected for the Y-axis. For improving the accuracy, it might be necessary to use a better accelerometer to improve the accuracy of speed estimation of the INS, because it is difficult to replace the Doppler sonar. 

#### 4.3.3. Evaluation of Position Estimation

**Figure 15:**
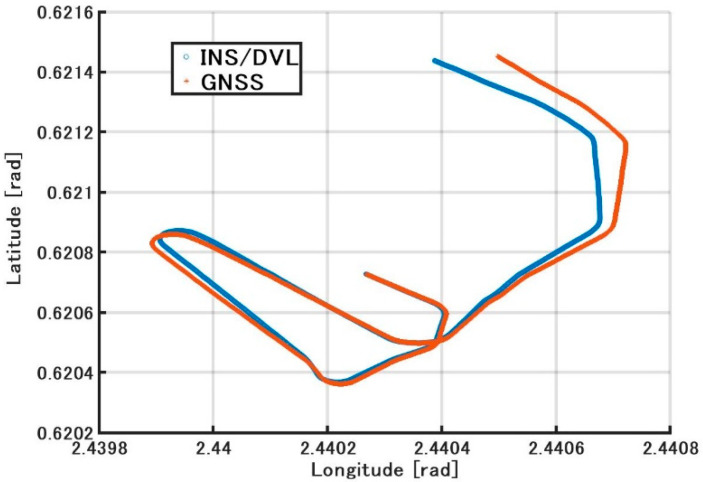
Trajectory with latitude and longitude.

**Figure 16:**
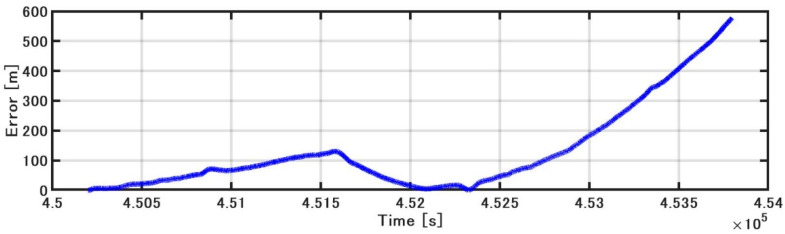
Horizontal error.

**Figure 17:**
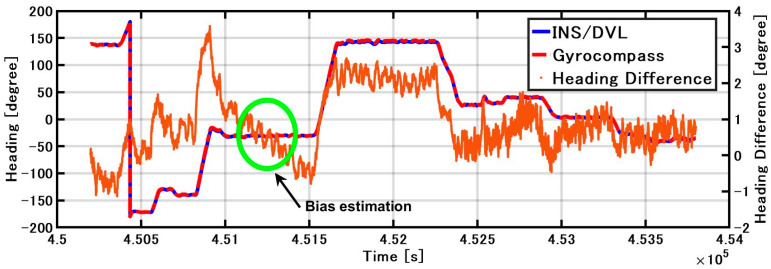
INS/DVL and gyrocompass heading angle and difference.

**Figure 18:**
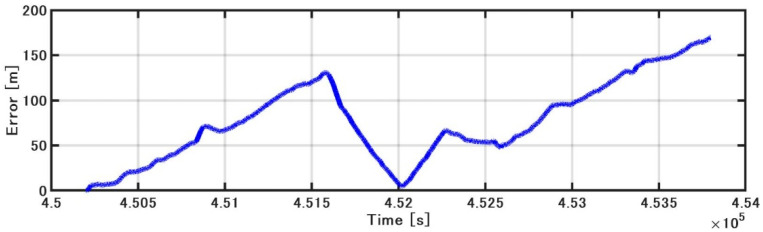
Horizontal error.

Figure 15 shows the position estimated by the INS/DVL and RTK-GNSS. Figure 16 shows the horizontal position difference between INS/DVL and RTK-GNSS. Figure 16 shows that the horizontal error was reduced because the angular velocity bias around the Z-axis was canceled out by the approximately 360° turn at around 4.52 × 10^5^ [s]. Subsequently, the position estimation error increased as well as the effect of the heading error shown in Figure 13, and the final error was 579 m. In this study, we used a gyrocompass as a reference. If we use the gyrocompass values to estimate the heading bias in the circled area in Figure 17 and then correct for the heading bias, we obtain the result in Figure 18, where the final horizontal error is 170 m. This post-processing method was only applied to the results of Figure 13 found to contain bias and is, therefore, not applicable as a real-time bias detection and correction method at this time. 

## 5. Discussion

In this section, the results of the two methods are summarized and briefly discussed. The first method used only the yaw-rate gyroscope of the IMU and DVL for 1 h dead-reckoning. The horizontal position error increased up to 943 m in a 1 h sailing duration. Here, two important points should be considered. The first is to estimate the initial direction at the beginning of the test. The GNSS-derived direction can be used for this purpose if the slip angle of the vessel is very small. The second is to estimate the temporal bias of the yaw-rate angle. This is very important in the use of IMUs because the sensor output drifts easily. Although temperature correction is also important, we did not correct the effect of the change in temperature because the largest difference in temperature in this test was 1°. In general, the temperature of the IMU inside a ship is not difficult to maintain. In summary, a cumulative horizontal error of approximately 1000 m per hour was achieved using only a low-cost IMU and the vessel’s standard DVL, even with several 90°–180° turns. This means that we could navigate the vessel without GNSS, due to spoofing or interference attacks for example, for 1 h with an accuracy of approximately 1000 m. The second method used three accelerometers and three gyroscopes of the IMU with the DVL. The approach of the second method differed from that of the first simple method in the following ways. First, the parameters of the AV for the target IMU were investigated thoroughly. Based on these estimated parameters, INS/DVL integration was conducted using EKF. The horizontal position error increased up to 579 m in the 1-h sailing duration. The accumulated error was reduced because all parameters and outputs of the IMU were used to estimate the direction and attitude of the vessel.

As mentioned in the Introduction, very few previous studies have used IMU and DVL to estimate the position of a ship. In a study where the GNSS outage was set for only 5 min [15], experiments were conducted using various KFs. A comparison of our results with that study is impractical because of the significantly different experimental timeframes. Considering the recent challenges posed by GNSS spoofing, it might be necessary to develop a system that can guarantee about 30 min to one hour without functional GNSS.

It is possible to improve the accuracy of the system we studied (method 2) by using FOG [6], but the installation cost of an INS system using FOG may be prohibitively high for merchant ships and small boats. Even the pure inertial mode of the very expensive iXblue PHINS IMU has a specification accuracy of 0.6 nm/h [29]. If we integrated a low-cost IMU with a DVL, we could achieve similar accuracy. Further improvements in accuracy are needed for discussions, such as IMO’s Standardization. According to International Maritime Organization guidelines, “Where a radionavigation system is used to assist in the navigation of ships in ocean waters, the system should provide positional information with an error not greater than 100 m with a probability of 95%” [30]. Since this criterion applies to a radio-navigation system, it does not specify time and cannot be directly applied to a system such as INS, in which the effect of error accumulation over time is large. However, we believe that this criterion can serve as a target value for the improvement of systems such as the one presented in this study.

We conducted another experiment similar to the one described in this paper on a different day with high waves in deeper water in which the Doppler sonar could not measure the ground speed. As mentioned in Section 4, the Y-axis of the Doppler sonar was found to be inaccurate. It is important to use the proper Doppler sonar and determine the limitations of the Doppler sonar for measuring the depths. Although this is true for both the first and second methods, if we can detect and correct the bias of the Z-axis angular velocity, we can improve the positional accuracy, as shown in Figure 18. However, real-time detection and correction of bias without GNSS will be an issue.

## 6. Conclusions

In this study, IMU/DVL integrated positioning was proposed as a back-up system when GNSS is unavailable, and its positioning accuracy was evaluated. Two different methods of dead-reckoning were evaluated. In the first, using only the yaw-rate gyroscope and DVL, the maximum horizontal error was 943 m in 1 h. Using the second, more sophisticated INS/DVL method, the maximum horizontal error was reduced to 579 m in 1 h. While this is a significant improvement, there is a limitation to accurate estimation using only an IMU with either method. Future improvements in positional accuracy may be made through detection and correction of the Z-axis angular velocity bias, and improved prediction or measurement of the slip angle. These two points are subject to further study.

In this study, we used a training ship with students on board. Although the students were aware that GNSS is the only device that can automatically estimate the position on a ship, few of them were aware that the vulnerability of GNSS is a concern and needs to be discussed [31]. We recognize that it is important in maritime education to promote this kind of research involving students.

## Figures and Tables

**Table 1:** Parameter estimation by Allan variance (AV).

	Static Bias (rad/s) (m/s^2^)	STD (rad/s) (m/s^2^)	Random Walk $(\mathrm{rad}/s\sqrt{Hz}$$)(m/s^{2}\sqrt{Hz}))$	Bias Instability (rad/s) (m/s^2^)
Gyro X	−7.825 × 10^−^^04^	3.088 × 10^−04^	4.00 × 10^−05^	2.63 × 10^−05^
Gyro Y	3.234 × 10^−03^	3.107 × 10^−04^	4.00 × 10^−05^	2.90 × 10^−05^
Gyro Z	2.202 × 10^−03^	3.307 × 10^−04^	4.30 × 10^−05^	2.67 × 10^−05^
Acc X	6.389 × 10^−02^	9.326 × 10^−03^	1.29 × 10^−03^	9.35 × 10^−04^
Acc Y	5.178 × 10^−01^	1.005 × 10^−02^	1.69 × 10^−03^	1.59 × 10^−03^
Acc Z	−9.940	9.255 × 10^−03^	1.40 × 10^−03^	1.20 × 10^−03^

**Table 2:** Summary of the converted input data.

	X-Axis	Y-Axis	Z-Axis
$b_{g}$	2.63 × 10^−5^ [rad/s]	2.90 × 10^−5^ [rad/s]	2.67 × 10^−5^ [rad/s]
$b_{f}$	9.34 × 10^−4^ [m/s^2^]	1.60 × 10^−03^ [m/s^2^]	1.20 × 10^−3^ [m/s^2^]
$S_{rg}$	0.966 × 10^−7^ [rad] (Equation (14.81) of [27])		
$S_{ra}$	0.101 × 10^−3^ [m/s] (Equation (14.81) of [27])		
$S_{bgd}$	0.115 × 10^−10^ [rad]	0.115 × 10^−10^ [rad]	0.115 × 10^−10^ [rad]
$S_{bad}$	0.435 × 10^−7^ [m/s]	0.261 × 10^−7^ [m/s]	0.435 × 10^−7^ [m/s]
$\tau_{g}$	60 [s]	60 [s]	60 [s]
$\tau_{f}$	60 [s]	100 [s]	60 [s]
$\tau_{s}$	1.0 [s]		

**Table 3:** Sensor information.

	GNSS	IMU		DVL
**Name**	Trimble SPS855	CSM-MG100		ATLAS DOLOG SYSTEM
**Frequency**	5 Hz	100 Hz		1 Hz
**Accuracy**	**Position**	**Gyro**	**Acceleration**	**Speed**
	<0.1 [m]	±0.01 [m/s^2^]	±0.00175 [rad/s]	0.01 [knot] or 0.2% of the measured value

**Table 4:** Specifications of the TG-5000 gyrocompass.

**Setting Time**	Within 2 h	**Accuracy on Scorsby Table**	Less than ±0.5°
**Setting Point Error**	Less than ±0.3°	**Repeatability of Setting Point**	Less than ±0.2°
**RMS Value**	Less than 0.1°	**Accuracy Under Environmental Variation**	Less than ±0.5°

**Table 5:** Specifications of the JCS7402-A.

	Digital Output
**Range**	±Roll: ±180°, Pitch: ±90°
**Resolution**	<0.1°
**Accuracy**	<±0.15° at input <±10° <± (0.2° + 1% of input) at input = ±10°~45°

## Data Availability

The data presented in this study are available on request from the corresponding author. The data are not publicly available due to the matter of the training ship. When a request is received for a reasonable reason, it can only be provided after explaining it to the relevant department and obtaining permission from all departments.
